# Significance of combined cyclosporine−prednisolone therapy and cyclosporine blood concentration monitoring for idiopathic membranous nephropathy with steroid-resistant nephrotic syndrome: a randomized controlled multicenter trial

**DOI:** 10.1007/s10157-013-0925-2

**Published:** 2013-12-23

**Authors:** Takao Saito, Masayuki Iwano, Koichi Matsumoto, Tetsuya Mitarai, Hitoshi Yokoyama, Noriaki Yorioka, Shinichi Nishi, Ashio Yoshimura, Hiroshi Sato, Satoru Ogahara, Hideki Shuto, Yasufumi Kataoka, Shiro Ueda, Akio Koyama, Shoichi Maruyama, Masaomi Nangaku, Enyu Imai, Seiichi Matsuo, Yasuhiko Tomino, The Refractory Nephrotic Syndrome Study Group

**Affiliations:** 1 General Medical Research Center, Faculty of Medicine, Fukuoka University, 7-45-1 Nanakuma, Jonan-ku, Fukuoka, 814-0180 Japan; 2 Division of Nephrology, Department of General Medicine, Faculty of Medical Sciences, University of Fukui, Fukui, Japan; 3 The University Research Center, General Science Institute, School of Medicine, Nihon University, Tokyo, Japan; 4 Department of Nephrology and Blood Purification, Saitama Medical Center, Saitama Medical University, Kawagoe, Japan; 5 Division of Nephrology, Kanazawa Medical University School of Medicine, Ishikawa, Japan; 6 Hiroshima Kidney Organization, Hiroshima, Japan; 7 Division of Nephrology and Kidney Center, Kobe University Graduate School of Medicine, Kobe, Japan; 8 Division of Nephrology, Department of Internal Medicine, Showa University Fujigaoka Hospital, Yokohama, Japan; 9 Division of Nephrology, Tohoku University Graduate School of Medicine, Sendai, Japan; 10 Division of Nephrology and Rheumatology, Faculty of Medicine, Fukuoka University, Fukuoka, Japan; 11 Department of Pharmaceutical Care and Health Sciences, Faculty of Pharmaceutical Sciences, Fukuoka University, Fukuoka, Japan; 12 Ueda Clinic, Chiba, Japan; 13 Department of Nephrology, Tsukuba Memorial Hospital, Ibaraki, Japan; 14 Department of Nephrology, Nagoya University Graduate School of Medicine, Nagoya, Japan; 15 Division of Nephrology and Endocrinology, University of Tokyo School of Medicine, Tokyo, Japan; 16 Nakayamadera Imai Clinic, Hyogo, Japan; 17 Division of Nephrology, Department of Internal Medicine, Juntendo University Faculty of Medicine, Tokyo, Japan

**Keywords:** Cyclosporine, Idiopathic membranous nephropathy, Steroid-resistant nephrotic syndrome, Once-a-day administration, Preprandial administration, Therapeutic drug monitoring

## Abstract

**Background:**

Combined treatment with cyclosporine microemulsion preconcentrate (CyA MEPC) and steroids has been widely used for idiopathic membranous nephropathy (IMN) associated with steroid-resistant nephrotic syndrome (SRNS). Recent studies have shown that once-a-day and preprandial administration of CyA MEPC is more advantageous than the conventional twice-a-day administration in achieving the target blood CyA concentration at 2 h post dose (C2). We designed a randomized trial to compare these administrations.

**Methods:**

IMN patients with SRNS (age 16–75 years) were divided prospectively and randomly into 2 groups. In group 1 (*n* = 23), 2–3 mg/kg body weight (BW) CyA MEPC was given orally once a day before breakfast. In group 2 (*n* = 25), 1.5 mg/kg BW CyA MEPC was given twice a day before meals. CyA + prednisolone was continued for 48 weeks.

**Results:**

Group 1 showed a significantly higher cumulative complete remission (CR) rate (*p* = 0.0282), but not when incomplete remission 1 (ICR1; urine protein 0.3–1.0 g/day) was added (*p* = 0.314). Because a C2 of 600 ng/mL was determined as the best cut-off point, groups 1 and 2 were further divided into subgroups A (C2 ≥600 ng/mL) and B (C2 <600 ng/mL). Groups 1A and 2A revealed significantly higher cumulative remission (CR + ICR1) (*p* = 0.0069) and CR-alone (*p* = 0.0028) rates. On the other hand, 3 patients with high CyA levels (C2 >900 ng/mL) in Group 1A were withdrawn from the study because of complications.

**Conclusion:**

CyA + prednisolone treatment is effective for IMN with associated SRNS at a C2 of ≥600 ng/mL. To achieve remission, preprandial once-a-day administration of CyA at 2–3 mg/kg BW may be the most appropriate option. However, we should adjust the dosage of CyA by therapeutic drug monitoring to avoid complications.

## Introduction

Idiopathic membranous nephropathy (IMN) is the most representative disease associated with steroid-resistant nephrotic syndrome (SRNS) in adults. Although the combination of steroids and immunosuppressants, e.g., cyclophosphamide (CPA) and chlorambucil, has been reported to induce and maintain remission in randomized controlled studies [1, 2], the beneficial effects remain controversial because of the harmful side-effects of the alkylating agents. Moreover, in our cohort study of 1,000 cases in Japan, combined treatment with steroids and CPA was not superior to steroid monotherapy [3]. Recently, cyclosporine (CyA), a calcineurin inhibitor, has been introduced as an effective agent for SRNS, and several randomized controlled trials (RCTs) on the combination of steroids and CyA showed significant remission rates [4–6].

However, it has been recognized that clinical response does not correlate well with the administration dose. Accordingly, careful attention to the CyA concentration in blood is essential for the optimization of therapy [7]. For this reason, the blood concentration of the drug was previously monitored at the trough level before administration (C0) because the absorption of CyA is highly affected by bile acid and other factors of absorption when the original CyA formulation was used orally [8]. The introduction of CyA microemulsion preconcentrate (MEPC) minimized the influence of bile acid and stabilized the absorption profile (AP) of CyA [9]. In a transplantation study, the area under the blood concentration–time curve up to 4 h after administration of CyA (AUC0–4) was believed to accurately express CyA absorption and sensitively predict the effect of CyA [10]. Moreover, the CyA blood concentration at 2 h post dose (C2) was recommended as the best surrogate single-sample marker for routine monitoring [10].

Recent studies have shown that once-a-day administration is more advantageous than the conventional twice-a-day administration, because the former provides an AP showing the peak blood concentration of CyA, which may facilitate the remission of SRNS and prevent chronic CyA nephrotoxicity [11, 12]. In addition, preprandial administration of CyA may be favorable for achieving a stable blood concentration because CyA is absorbed without the influence of food ingestion [12, 13]. However, there is no evidence that such therapeutic strategies contribute to the remission of SRNS.

In this study, we designed a prospective, open-label randomized trial to compare the effect of preprandial once-a-day administration of CyA with that of conventional twice-a-day administration for IMN with associated SRNS. Blood CyA concentrations at C0 and C2 were also evaluated during treatment.

## Methods

This study was registered at the University Hospital Medical Information Network-Clinical Trials Registry (UMIN-CTR) under trial identification no. UMIN C000000369 and was approved by the Clinical Study Review Board at Fukuoka University Hospital (approval no. 03-129). The study was conducted in accordance with the principles of the declaration of Helsinki. Written informed consent was obtained before patient enrollment and after a thorough explanation of the trial’s objectives, duration, and structure. The availability of alternative drugs, the possibility of adverse reactions, privacy measures, and the voluntary nature of the trial, including the right to withdraw without repercussions, were all carefully explained. The institutional review boards at the collaborating institutions also approved the protocol when requested.

### Patients

SRNS patients (age 16–75 years) with IMN diagnosed by renal biopsy were enrolled through computerized registration from kidney centers in Japan between 2004 and 2007. Membranous nephropathy secondary to systemic diseases, e.g., diabetic nephropathy and collagen diseases, were excluded at registration. Nephrotic syndrome (NS) was defined according to the standard criteria in Japan [3]—(1) urine protein (UP) excretion >3.5 g/day; (2) serum albumin <3.0 g/dL or serum total protein <6.0 g/dL; (3) presence of edema; and (4) total cholesterol >250 mg/dL. At least the first and second criteria were necessary for the diagnosis. SRNS was determined when patients did not achieve complete remission (CR) or incomplete remission (ICR) 1 (as described in ‘Clinical assessment’ section) after 4 weeks of prednisolone (PSL) therapy at 40–60 mg/day. The inclusion and exclusion criteria are listed in Table 1.

**Table 1:** Inclusion and exclusion criteria

Inclusion criteria
1. Age between 16 and 75 years
2. UP >3.5 g/day and serum albumin level <3.0 g/dL
3. PSLalone treatment for >4 weeks did not decrease UP into <1 g/day
4. Membranous nephropathy was diagnosed by renal biopsy.
5. No history of treatment with CyA-MEPC before registration
6. Informed consent form voluntarily signed by the participant
Exclusion criteria
1. Patients with creatinine clearance <50 mL/min or serum creatinine >2 mg/dL
2. Patients that received other immunosuppressants within 1 month before the study commencement
3. Patients treated with nephrotoxic and hyperkalemic agents during the study period
4. Patients with a malignant tumor or a history of a recurrent malignant tumor
5. Patients with hypertension uncontrolled with antihypertensive drugs
6. Patients with malabsorption syndrome, cerebral dysfunction, or epilepsy
7. Patients with hyperkalemia or hyperuricemia
8. Patients with a severe cardiac, hepatic, or pancreatic disease
9. Patients currently pregnant, suspected to be pregnant, or nursing
10. Patients with an infectious complication and not eligible for treatment with immunosuppressants
11. Patients with a history of hypersensitivity to CyA-MEPC
12. Patients determined to be inappropriate for participation in the study by an investigator

*UP* urine protein, *PSL* prednisolone, *CyA-MEPC* cyclosporine microemulsion preconcentrate

Renal histology was assessed according to the following 5 parameters—presence of global sclerosis and segmental sclerosis in glomeruli, severity of tubulointerstitial changes, occurrence of vascular lesions, and ultrastructural stage of glomerular lesions according to the criteria of Ehrenreich and Churg [14]. These changes were estimated semiquantitatively as we previously reported [3], and compared between groups.

### Study design

Patients were divided prospectively and randomly into 2 groups (groups 1 and 2). Combined administration of PSL and CyA MEPC was continued for 48 weeks. PSL was initially prescribed at 40 mg/day and tapered gradually to <10 mg/day by 48 weeks. In group 1, CyA MEPC was given orally once a day before breakfast at 2–3 mg/kg body weight (BW). In group 2, CyA MEPC was given twice a day before meals at 1.5 mg/kg BW each. Other agents, including antihypertensive, antidyslipidemic, and anticoagulant drugs, were allowed unless their combination with CyA was contraindicated. Biochemical data, including total protein, albumin, urea nitrogen, creatinine, and total cholesterol in serum, and 24-h UP, were assayed at 0, 4, 8, 12, 24, 36, and 48 weeks.

### CyA treatment and monitoring

To determine the AP of CyA in each patient, blood CyA concentrations from 0 to 4 h (C0–C4) were assayed within 1 month of treatment, and the AUC0–4 (ng h/mL) was calculated. The linear trapezoid formula was used with C0 to C4. Then, C0 and C2 were repeatedly assayed during the treatment period.

In group 1, CyA was started at 2 mg/day and dose adjustments were made to achieve a C0 of 80–120 ng/mL and C2 of 800–1,000 ng/mL. The CyA dose was increased to a maximum of 3 mg/day when the target C0 and C2 were not achieved. In contrast, the dose was reduced when C0 and C2 exceeded the target levels. In group 2, adjustments were also made so as not to exceed C0 and C2 by 120 and 1,000 mg/dL, respectively. In the maintenance phase after remission, the dose was adjusted so as not to exceed C0 and C2 by 80 and 800 mg/dL, respectively. The whole blood concentration of CyA was measured by radioimmunoassay or by the fluorescence polarization immunoassay methods of SRL Co., Japan, or the biochemical laboratory of each kidney center. The average C0 and C2 during the treatment period before remission were used for the comparison of outcomes.

### Clinical assessment

Clinical assessment of treatment outcomes was performed on the basis of changes in proteinuria and renal function, partly modified from the previous criteria in Japan [3]. Briefly, CR was defined when the UP was <0.3 g/day. ICR was defined as the resolution of NS but with continuing overt proteinuria, and was divided into 2 grades—ICR1 and ICR2 for UP of 0.3–1.0 and 1.0–3.5 g/day, respectively. No response (NR) was defined as the persistence of NS. Since patients with ICR1 showed a favorable prognosis almost equal to CR in a previous study [3], we considered CR + ICR1 as remission. For renal function, 3 categories were defined according to serum creatinine concentration—(1) normal renal function <1.5 mg/dL; (2) renal insufficiency 1.5–3.0 mg/dL; and (3) end-stage renal disease >3.0 mg/dL.

### Statistical analysis

Values were given as mean ± SE or median (interquartile range). Differences in clinical characteristics between the 2 groups were evaluated with Student’s *t* test and Mann–Whitney *U* test for continuous variables and Fisher’s exact test for categorical variables. The incidence of remission (CR + ICR1) or CR was compared using Fisher’s exact test. Time to remission or CR curves for the therapy groups were estimated using the Kaplan–Meier technique, and the curves were compared using the log-rank test.

The effects of blood CyA concentrations and clinical variants for the incidence of remission were examined using logistic regression analysis. The variants that affected serum CyA concentrations were examined using multiple regression analysis.

Receiver operating characteristic (ROC) curve analysis was used to test the prognostic value of serum CyA concentrations (average C0 and C2) and to determine the best cut-off for the prediction of CR.

All statistical analyses were performed using SPSS for Windows version 18.0 (SPSS Japan Inc., Tokyo, Japan).

## Results

**Fig. 1:**
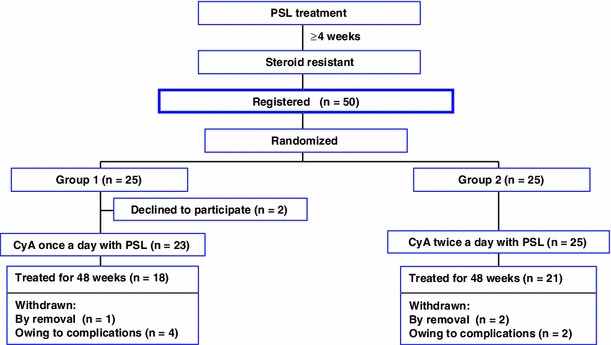
Flowchart of the study design: enrollment of patients and treatment assignment

The flowchart of the study design regarding enrollment of patients and treatment assignment is shown in Fig. 1.

### Patients

Fifty patients in 30 kidney centers in Japan were registered according to the inclusion criteria, from April 2004 to December 2007, and 25 patients each were randomly enrolled in the once-a-day (group 1) and twice-a-day (group 2) administration groups. However, 2 patients in group 1 declined to participate in this study before CyA treatment. Consequently, 23 and 25 patients were treated with PSL and CyA in groups 1 and 2, respectively. The baseline clinical characteristics of all patients are summarized in Table 2. There was no significant difference in each item between the 2 groups. Five parameters of renal histology estimated semiquantitatively did not show significant differences between groups (data not shown).

**Table 2:** Baseline characteristics of patients with idiopathic membranous nephropathy

Characteristic	Group 1 (*n* = 23)	Group 2 (*n* = 25)	*p*
Sex (male/female)	16:7	17:8	0.91
Age	56 (19–70)	57 (39–70)	0.48
Urine protein (g/day)	3.5 (1.8–10)	3.8 (1.0–6.5)	0.63
Serum levels			
Urea nitrogen (mg/dL)	14 (8–24)	15 (9–33)	0.54
Creatinine (mg/dL)	0.8 (0.5–1.2)	0.8 (0.6–1.6)	0.84
Total protein (g/dL)	4.7 (3.9–6.2)	4.7 (3.6–5.6)	0.15
Albumin (g/dL)	2.7 (2.2–3.5)	2.6 (1.5–3.3)	0.09
Total cholesterol (mg/dL)	314 (229–617)	298 (213–853)	0.52

Age and laboratory data are shown as median (interquartile range)

The *p* values were evaluated by Fisher's exact test for sex and Mann–Whitney *U* test for the others

A previous study on IMN treated with a combination of PSL and CyA (2–3 mg/kg/day, twice-a-day) showed a 35 % CR ratio at the 12-month course [6]. However, there were no data for once-a-day administration. Nevertheless, the sample size (groups 1 and 2: *n* = 23 and *n* = 25, respectively) was sufficient to detect a significant difference (*α* = 0.05, 2-sided) on the basis of 0.8 power according to Fisher’s exact test when once-a-day administration is twice as effective (CR ratio 70 %) than twice-a-day administration. Therefore, we stopped the registration at the end of 2007.

As shown in Table 3, during the treatment, 1 patient in group 1 and 2 patients in group 2 were transferred to another hospital and could therefore not further participate in the study. Four patients in group 1 and 2 patients in group 2 were withdrawn because of complications and noncompliance. Finally, 18 and 21 patients in groups 1 and 2 completed the study for 48 weeks.

**Table 3:** Withdrawn patients

Group	Withdrawal period (weeks)	Reason	Average C2 (ng/mL)
Group 1 (*n* = 5)	9	Nausea	1042
	10	Uncontrolled CyA level	1200
	12	Liver dysfunction	750
	12	Pneumonia	936
	40	Removal	
Group 2 (*n* = 4)	8	Brain tumor^a^	693
	36	Noncompliance	813
	10	Removal	
	12	Removal	

^a^May not be related to CyA administration

### Responses in the once-a-day and twice-a-day administration groups

The response around 6 months is important to determine the initial effect of CyA treatment as shown in RCTs and guidelines [4, 5, 15–17]. In the intention-to-treat analysis, 10 of 23 patients (43.5 %) in group 1 and 2 of 25 patients (8.0 %) in group 2 achieved CR at 24 weeks. This yielded a significant difference between groups in Fisher’s exact test (*p* = 0.0078). In group 1, two other patients achieved CR at 8 and 12 weeks, respectively; however, the first patient relapsed into ICR2 by 24 weeks and the second was withdrawn thereafter because of liver dysfunction. ICR1 occurred in 1 and 10 patients in groups 1 and 2, respectively. In total, 11 (47.8 %) patients in group 1 and 12 (48.0 %) in group 2 achieved remission (CR + ICR1) (*p* = 1.000).

**Fig. 2:**
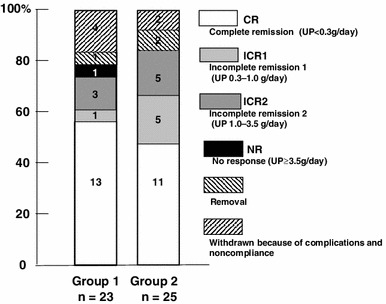
Remission and withdrawal rates of groups 1 and 2 at 48 weeks. Patients were divided according to CyA administration frequency—once a day (group 1) or twice a day (group 2). In each therapeutic response, there was no significant difference

Between 24 and 48 weeks, more patients achieved CR in both groups, but a few patients with CR relapsed conversely. At 48 weeks, 13 of 23 patients (56.5 %) in group 1 and 11 of 25 patients (44.0 %) in group 2 were in CR, and 14 of 23 (60.9 %) in group 1 and 16 of 25 (64.0 %) in group 2 were in CR + ICR1 (Fig. 2). For each therapeutic response, there was no significant difference between groups. In the per-protocol analysis, similar results were statistically obtained at 24 and 48 weeks.

**Fig. 3:**
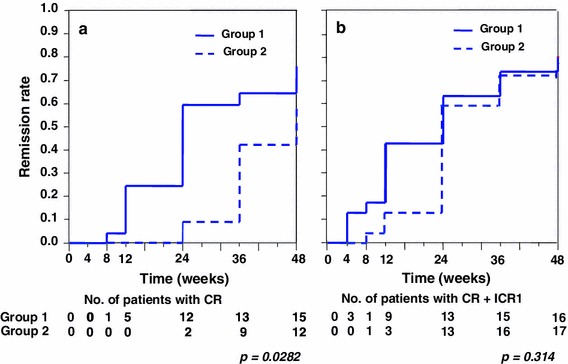
Probability of cumulative complete remission (CR) (**a**) and CR + incomplete remission 1 (ICRI) (**b**) for patients treated with PSL and CyA. Group 1 showed a significantly higher rate of CR (**a**) but not of CR + ICRI (**b**) compared with group 2

However, the time-to-remission curve analyzed using the Kaplan–Meier technique revealed a significant deference in cumulative CR rate (*p* = 0.0282; Fig. 3a) but not in cumulative CR + ICR1 rate (*p* = 0.314, Fig. 3b).

### Assessment of clinical parameters

After CyA + PSL treatment, the levels of UP, serum albumin, and serum total cholesterol significantly improved in both groups; however, there were no significant differences in each parameter between the 2 groups. Serum creatinine level slightly increased in both groups but was not significant. Two patients in each group exhibited a doubling of serum creatinine, around 2 mg/dL, at 48 weeks, although the levels were within the reference range at the start of treatment.

At baseline, only 1 patient had mild hypertension in group 2 (155/89 mmHg), but the blood pressure normalized later. At the final observation, another patient in group 2 showed mild hypertension (150/88 mmHg). No patient had CyA-induced hypertension in either group. As the supportive therapy for MN, angiotensin II receptor blockers (4 and 2 patients in groups 1 and 2, respectively) and angiotensin-converting enzyme inhibitors (one in group 1) and a combination of both (one in each group) were administered. However, these drugs did not produce any adverse effects including hyperkalemia.

Although four patients in groups 1 and 2 showed mild hyperglycemia by steroids treatment, respectively, this did not have any serious influences on the results.

### Blood CyA concentrations

**Fig. 4:**
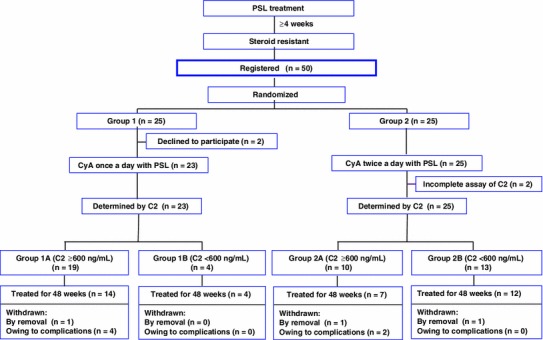
Flowchart of the study design: assignment by CyA blood concentrations at 2 h post dose (C2)

The flowchart of the study design regarding assignment by blood CyA concentrations at 2 h post dose (C2) is shown in Fig. 4.

#### Absorption profiles of CyA in groups 1 and 2

There were significant differences in AUC0–4 between groups (group 1 vs group 2: 3678 ± 181 vs 2506 ± 164 ng h/mL, *p* < 0.0001). In comparisons between AUC0–4 and CyA concentrations at each time point (C0–C4), C2 was most strongly correlated with AUC0–4 in the total patients (*r* = 0.032, 0.609, 0.780, 0.654, 0.579 for C0, C1, C2, C3, C4, respectively).

#### Average C0 and C2 and the cut-off level for CR

**Fig. 5:**
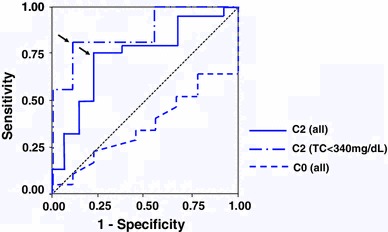
Receiver operator characteristic (ROC) curves for serum CyA concentration. The optimal cut-off level of C2 for CR was determined to be 615 ng/mL (sensitivity 75.6 %, specificity 76.9 %) and 598 ng/mL (sensitivity 81.3 %, specificity 88.9 %) (*arrows*), using the ROC curve drawn from the average C2 of all cases and the cases <340 mg/dL of total cholesterol treated for 48 weeks in groups 1 and 2, respectively

The average C0 and C2 during treatment were significantly correlated with the C0 and C2 at the AP, respectively (C0: *r* = 0.516, *p* = 0.0036; C2: *r* = 0.638, *p* = 0.0001). The average C2 in group 1 was significantly higher than in group 2; however, the average C0 in group 1 was significantly lower than in group 2. Only C2 significantly predicted CR in logistic regression analysis based on C0, C2, age and baseline laboratory factors related to renal function and NS. Moreover, a multiple regression model showed that C2 was not significantly related to other variants as above. ROC curves were drawn to detect the optimum cut-off level of the average C2 or C0 for CR (Fig. 5). Using all data of the cases treated for 48 weeks in groups 1 and 2 (*N* = 37), the area under ROC curves were 0.731 ± 0.089 (95 % CI 0.557–0.905, *p* = 0.022) for C2 and 0.373 ± 0.109 (95 % CI 0.156–0.587, not significant) for C0. From these results, the optimum cut-off point for C2 was determined to be 615 ng/mL (sensitivity 75.0 %, specificity 76.9 %); however, C0 was inappropriate to predict remission. Using the data of group 2 alone (*N* = 19), similar results were obtained. Namely, the AUCs were 0.802 ± 0.101 (95 % CI 0.604–1.000, *p* = 0.025) for C2 and 0.444 ± 0.158 (95 % CI 0.135–0.754, not significant) for C0, and the cut-off point for C2 was determined to be 598 ng/mL (sensitivity 66.7 %, specificity 100 %). When the data of C2 were limited to the cases <340 mg/dL of total cholesterol (*N* = 25), the AUCs were greater (0.868 ± 0.072, 95 % CI 0.712–1.000, *p* = 0.003) and the cut-off point 598 ng/mL was more accurately provided (sensitivity 81.3 %, specificity 88.9 %).

### Relationship between blood CyA concentration and treatment responses

**Fig. 6:**
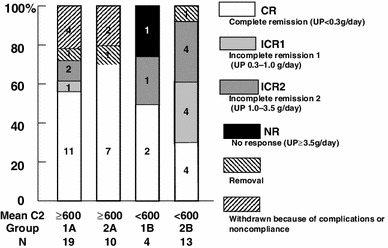
Remission and withdrawal rates of groups 1A, 1B, 2A, and 2B at 48 weeks. Patients were divided into groups 1 and 2 according to administration frequency and then subdivided into subgroups A (C2 ≥600 ng/mL) and B (C2 <600 ng/mL). There was a significant difference in CR between groups A and B (*p* = 0.018, per-protocol analysis)

**Fig. 7:**
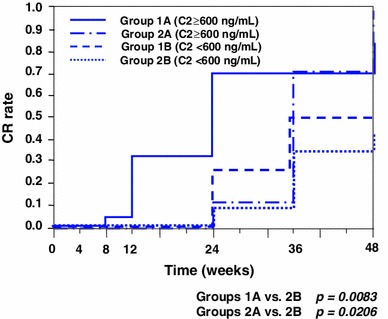
Probability of cumulative CR for patients treated with PSL and CyA. Groups 1A and 2A showed significantly higher remissions compared with group 2B

**Fig. 8:**
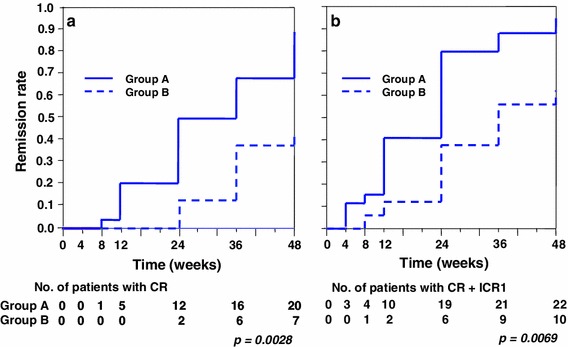
Probability of cumulative CR (**a**) and CR + ICRI (**b**) for patients treated with PSL and CyA. Group A (1A + 2A) showed a significantly higher remission rate compared with group B (1B + 2B) in both analyses

Patients in groups 1 and 2 were further divided into subgroups A (C2 ≥600 ng/mL) and B (C2 <600 ng/mL) because the ROC showed that the optimal cut-off point of C2 was approximately 600 ng/mL. The number of patients in groups 1A, 1B, 2A, and 2B was 19, 4, 10, and 13, respectively (Fig. 6). Most of the patients in groups 1A and 2A achieved CR. Among these 4 groups, groups 1A and 2A showed significantly higher cumulative CR ratios than group 2B for 48 weeks; group 1B was excluded because of the statistically insufficient number of patients (Fig. 7). Meanwhile, there was no significant difference between groups 1A and 2A. Groups 1A and 2A, consisting of all patients with C2 ≥ 600 ng/mL, also showed a significantly higher cumulative ratio of not only CR (*p* = 0.0028, Fig. 8a) but also CR + ICRI (*p* = 0.0069, Fig. 8b) than groups 1B and 2B (C2 <600 ng/mL).

Four patients in group 1A were withdrawn from the study because of complications that may be related to CyA administration (Table 3). In 3 of these 4 patients, C2 was >900 ng/mL, although there was no significant difference in C2 between these 4 patients and the other 21 patients in group 1A.

## Discussion

The combined administration of CyA with steroids has been reported to be useful for the treatment of IMN with associated SRNS [5, 6, 18–20]. However, only a few randomized controlled trials have succeeded in clarifying this benefit [5, 6]. In the current randomized trial, we attempted to develop a more efficient strategy for CyA treatment by preprandial once-a-day administration. The effect of this method was significant for cumulative CR rate during 48 weeks using the Kaplan–Meier technique when compared with twice-a-day administration, but not for CR incidences at 48 weeks in the Fisher’s exact test. The discrepancy of the results might be influenced by the relapsing cases because these were included in cumulative CR cases in the Kaplan–Meier technique. On the other hand, it was possible that scattered distribution of blood CyA concentrations in both groups might obscure the effect, although C2 in group 1 was significantly higher than group 2.

ROC curve analysis was performed to assess the predictive value of blood CyA concentration for the outcome of NS. In comparison with C0, only C2 was available for predicting CR (Fig. 5). Interestingly, the predictive value of C2 was more enhanced when the hypercholesterolemic cases were excluded (Fig. 5). This study may demonstrate for the first time that hyperlipidemia in NS prevents CyA treatment, although the affinity of CyA to lipoproteins has been studied in transplantation [21, 22].

The optimal cut-off points for C2 were calculated as 615 and 598 ng/mL in all patients and in group 2, respectively. As these results suggest that CyA might be effective for IMN when C2 is approximately >600 ng/mL, we divided each group into subgroups A (C2 ≥600 ng/mL) and B (C2 <600 ng/mL).

Among these 4 subgroups, groups 1A and 2A showed significantly higher cumulative CR and CR + ICRI rates. Accordingly, regardless of whether the administration is once or twice a day, CyA blood concentration is a highly sensitive marker for the remission of NS. However, once-a-day administration seems to be more favorable because most of group 1 patients showed higher C2 concentrations. On the other hand, 3 patients in group 1A withdrawn from the study owing to complications showed an average C2 of >900 mg/dL, although there was no significant difference in C2 between the withdrawn patients and the remaining 21 patients in group 1A. Therefore, we think that the optimal strategy of CyA treatment is to maintain C2 between 600 and 900 ng/mL by preprandial once-a-day administration.

CyA is known to have a narrow therapeutic range of blood concentration. However, there is no study showing the relationship between drug monitoring and long-term outcomes in IMN, and C0 has been used as a standard parameter to determine the optimal dose of CyA without any evidence. Recently, transplantation studies [10, 23, 24] have shown that the AP of CyA-MEPC is stable and C2 is more reliable for 1-spot monitoring than C0 in correlation with AUC0–4. From this viewpoint, Levy et al. [28], according to the international consensus, suggested 1,400–1,600 ng/mL as the effective C2 in the early phase of renal transplantation. However, some authors have reported [26, 27] that the optimal C2 for Asian recipients is approximately 1,000 ng/mL. In NS, to achieve such an effective level of C2, a few studies have confirmed that preprandial and/or once-a-day administration was superior to the conventional twice-a-day administration [11–13].

To date, it has been assumed that the immunosuppressive effect of CyA results from the inhibition of the nuclear factor of activated T-cell signaling [28]. However, the remission of NS related to the CyA blood concentration could not be completely explained by the immunosuppressive mechanism. Faul et al. [29] demonstrated that CyA blocks the calcineurin-mediated dephosphorylation of synaptopodin in podocytes, thereby preserving the phosphorylation-dependent synaptopodin–14-3-3beta interaction. As a result, this direct effect of CyA on podocytes may contribute to the prompt reduction of UP, and prove the significance of CyA blood concentration monitoring on the therapeutic effect for NS. As it has been reported that steroids also directly preserve the function of podocytes [30, 31], the interaction between PSL and CyA in podocytes may play a pivotal role in the induction of remission in NS, when these agents are combined.

In the KDIGO (Kidney Disease: Improving Global Outcomes) clinical and practice guideline published in 2012 [15], the initial use of CPA with steroids was preferably recommended on the basis of evidence which was accumulated from many RCTs for over several decades. As mentioned above, however, the combined use of CyA with steroids has been recognized worldwide and was recently recommended by the Cyclosporin in Idiopathic Nephrotic Syndrome working group [16]. Moreover, the guidelines for the treatment of nephrotic syndrome in Japan [17] recommend combination treatment with steroids and CyA as the first choice for IMN because of at least 2 reasons. One is, as mentioned above, that our cohort study of 1,000 cases did not show the superiority of steroids + CPA over steroid monotherapy [3]; the other reason is that the risks of CPA use, e.g., neoplasia, agranulocytosis, and viral hepatitis, seem to be more fatal than those of CyA use, e.g., nephrotoxicity and hypertension. The current study shows that improved administration and drug monitoring are useful for increasing the benefits and decreasing the risks of CyA treatment, and may support the recommendations in the Japanese guidelines [17].

In our study, blood CyA concentration was measured by radioimmunoassay or monoclonal fluorescence polarization immunoassay. These methods are known to show 10–20 % higher levels of CyA than high-performance liquid chromatography (HPLC) as the gold standard [7] because nonspecific metabolites influence the assays [32]. On the other hand, affinity column-mediated immunoassay (ACMIA) was recognized to be comparable to HPLC [32–34] and has been widely used. Accordingly, our data should be corrected to lower values if the CyA concentration is measured by a new method such as ACMIA.

In conclusion, CyA combined with PSL is effective for the treatment of IMN associated with NS when the average C2 is >600 ng/mL. To achieve this concentration and induce remission, preprandial once-a-day administration of CyA at 2–3 mg/kg with PSL may be the most appropriate option. However, high blood CyA concentrations >900 ng/mL may frequently cause adverse effects and prevent the administration continuing. To avoid this, we should adjust the dosage of CyA by therapeutic drug monitoring.

## Appendix

The following members organized the trial:

Organizer: Takao Saito.

Protocol Committee: Hiroshi Sato, Shinichi Nishi, Tetsuya Mitarai, Koichi Matsumoto, Ashio Yoshimura, Hitoshi Yokoyama, Masayuki Iwano, Noriaki Yorioka, and Takao Saito.

Assessment Committee: Yasuhiko Tomino, Akio Koyama, and Shiro Ueda.

Statistics Committee: Yasufumi Kataoka, Hideki Shuto, and Satoru Ogahara.

Advisory Committee: Seiichi Matsuo and Enyu Imai, Masaomi Nangaku, and Shoichi Maruyama.

The following investigators participated in the trial:

Asahikawa Red Cross Hospital: Toshiya Ishiguro; Tohoku University: Hiroshi Sato; Fukushima Medical University: Masaaki Nagamichi; Saitama Medical University: Tetsuya Mitarai, Osamu Matsumura and Masaru Yoshikawa; Nihon University: Koichi Matsumoto and Takayuki Fujita; Showa University Fujigaoka Hospital: Ashio Yoshimura; Juntendo University: Yasuhiko Tomino and Yukihiko Takeda; The Jikei University: Yoichi Miyazaki; The Jikei University Kashiwa Hospital: Makoto Ogura and Akihiko Hamaguchi; Tokyo Women’s Medical University: Minako Koike; Tokyo Women’s Medical University Medical Center East: Masami Yoneda; Toho University: Sonoo Mizuiri; Teikyo University: Shunya Uchida; Hamamatsu University School of Medicine: Taro Misaki, Takehiko Miyaji, and Hideo Yasuda; Fujita Health University: Satoshi Sugiyama; Yokkaichi Municipal Hospital: Isao Ito; Mie University: Shinsuke Nomura; Osaka University: Enyu Imai; Nara Medical University: Hideo Shiiki and Masayuki Iwano; Kitano Hospital: Eri Muso and Toshiyuki Komiya; Niigata University: Shinichi Nishi; Kanazawa University: Hitoshi Yokoyama, Kiyoki Kitagawa and Takashi Wada; Kouseiren Takaoka Hospital: Miho Shimizu; Okayama University: Yohei Maeshima; Hiroshima University: Noriaki Yorioka and Takao Masaki; Kawasaki Medical School: Takehiko Tokura; Fukuoka University: Takao Saito and Satoru Ogahara; Miyazaki University: Seiichiro Hara; Kurume University: Keisuke Kono; Kyushu University: Kazuhiko Tsuruya.
